# “Liaisons dangereuses”: The invasive red‐vented bulbul (*Pycnonotus cafer*), a disperser of exotic plant species in New Caledonia

**DOI:** 10.1002/ece3.4140

**Published:** 2018-08-24

**Authors:** Martin Thibault, Felix Masse, Aurore Pujapujane, Guillaume Lannuzel, Laurent Bordez, Murray A. Potter, Bruno Fogliani, Éric Vidal, Fabrice Brescia

**Affiliations:** ^1^ Institut Agronomique néo‐Calédonien (IAC) Equipe ARBOREAL (AgricultuRe BiOdiveRsité Et vAlorisation) Païta New Caledonia; ^2^ Wildlife and Ecology Group School of Agriculture and Environment Massey University Palmerston North New Zealand; ^3^ Faculté des arts et des sciences Université de Montréal Montréal QC Canada; ^4^ Institut Méditerranéen de Biodiversité et d'Ecologie marine et continentale (IMBE) Aix Marseille Université CNRS, IRD, Avignon Université Centre IRD Nouméa Nouméa Cedex New Caledonia

**Keywords:** conservation, invasive bird, island, plant community, seed dispersal effectiveness

## Abstract

The biodiversity hotspot of New Caledonia hosts high levels of endemism (74% of flora) that is threatened increasingly by climate change, habitat reduction, and invasive species. The fruit‐eating red‐vented bulbul (*Pycnonotus cafer*) is currently invading the main island of the archipelago, and its recent dispersal out of urbanized habitats raises questions about its potential to disperse noxious plant seeds along urban corridors and beyond. Indeed, the red‐vented bulbul is considered a vector of several introduced plant species in its alien range including *Miconia calvescens*,* Lantana camara*, and *Schinus terebinthifolius*. We conducted a quantitative assessment of the bulbul's fruits consumption by analyzing the gut contents of shot birds. We estimated gut passage times for four species of fruit found in gut contents (*S. terebinthifolius*,* Myrtastrum rufopunctatum*,* Passiflora suberosa*, and *Ficus prolixa*) and tested the effects of bird digestion on seed germination rates for two species. Finally, we monitored the movements of individual VHF radio‐tagged red‐vented bulbuls. All of the consumed fruit species we identified here have red fleshy diaspore, including fruit of the shrub *M. rufopunctatum* that occurred frequently (9.6%) in bulbul gut samples. Median gut passage times were short (15–41 min), corresponding to short‐distance seed transportation (77–92 m). The effect of gut passage was positive for the germination of the invasive *S. terebinthifolius* and negative for the endemic *M. rufopunctatum*, suggesting a potential bias in the contribution to the dispersal toward alien species. This study provides the first integrated assessment of mechanisms involved in the seed dispersal effectiveness of this high‐concern invasive bird species that is expected to face similar plant communities in most of its alien range in tropical islands. More generally, our results enhance knowledge of synergies between non‐native frugivores and plant species dispersal.

## 1 INTRODUCTION

Construction, transportation, trade, and other human activities modify landscape structure, change plant and animal communities, drive changes in distribution patterns, and accelerate the rate of non‐native species dispersal leading to increasing biological invasions (Gosper, Stansbury, & Vivian‐Smith, 2005; Haddad et al., 2015; Hulme, 2009; Kokko & López‐Sepulcre, 2006; McConkey et al., 2012; Ramaswami, Kaushik, Prasad, Sukumar, & Westcott, 2016; Richardson et al., 2000; Smart et al., 2006). Dispersal of human cultures, together with animals and plants, is a key factor contributing to the current world biodiversity crisis (Ceballos et al., 2015). Thereby, dispersal has been explored from a variety of perspectives including its relevance to conservation biology (Levey, Silva, & Galetti, 2002; Primack & Miao, 1992; Trakhtenbrot, Nathan, Perry, & Richardson, 2005), restoration ecology (Bakker, Poschlod, Strykstra, Bekker, & Thompson, 1996; Ribeiro da Silva et al., 2015) and landscape ecology (Bacles, Lowe, & Ennos, 2006; Carlo & Morales, 2008).

An increasing number of studies investigate potential impacts of non‐native frugivorous species on plant dispersal in newly colonized ecosystems. Interactions between non‐native and native species are complex, but so are interactions among introduced species (Parker, Burkepile, & Hay, 2006; Relva, Nunez, & Simberloff, 2010). This is encapsulated in Simberloff and Von Holle's (1999) “invasional meltdown” hypothesis that postulates that mutualistic interactions between invaders can facilitate secondary invasions (Green et al., 2011). This phenomenon has been demonstrated widely in island territories (Bourgeois, Suehs, Vidal, & Médail, 2005; Davis, O'Dowd, Mac Nally, & Green, 2009; Traveset & Richardson, 2006), with positive interactions having been reported between introduced plants and birds (MacFarlane, Kelly, & Briskie, 2016; Traveset & Richardson, 2014).

How a frugivorous species contributes to the dispersal of plant species can be explored in a variety of ways. Direct observations help determine species’ diets and identify apparent close interactions between animal and plant species (Sherman & Fall, 2010), and excreta or gut content analysis can provide quantitative data to confirm observational assessments (Spotswood, Meyer, & Bartolome, 2012). Gut passage times can be used to predict dispersal distances and are often used in associated with radio tracking or global positioning system (GPS) data (Weir & Corlett, 2007), and germination tests can be used to determine how seed viability is enhanced or reduced by passage through a gut (Mokotjomela, Hoffmann, & Downs, 2015; Samuels & Levey, 2005). To predict the migration rates of plants along fragmented habitats, particularly those depending on few frugivore vectors, information on seed dispersal distance could be very useful (Pearson & Dawson, 2005).

Aslan and Rejmanek (2012) suggested that non‐native plant species that exhibit characters of native plants might outcompete native plants for attracting dispersers. They backed this up with reference to case studies where native dispersers preferred native plant‐of‐original characteristics, while non‐native birds such as the common starling (*Sturnus vulgaris*) preferred fruits of non‐native plant species. Preference for non‐native plant species has also been postulated for the introduced red‐vented bulbul (*Pycnonotus cafer*) in French Polynesia (Spotswood, Meyer, & Bartolome, 2013). Preferential seed dispersal of non‐native plant species by invasive passerines highlights an urgent need for quantitative assessments of the dispersal capacity of non‐native frugivorous species, especially in areas of high conservation value such as the world's biodiversity hotspots (Mittermeier, Turner, Larsen, Brooks, & Gascon, 2011).

New Caledonia is a tropical archipelago located in the South Pacific Ocean. Its geology and geographic isolation have produced unique ecosystems and high levels of endemism (74% for flora) (Cluzel, Aitchison, & Picard, 2001; Isnard, L'huillier, Rigault, & Jaffré, 2016; Munzinger et al., 2016). New Caledonia hosts 3,060 species of flowering plants, including an important metallophytic flora (Harrison & Rajakaruna, 2011), making the archipelago a terrestrial biodiversity hotspot (Myers, 2003). Its unique biodiversity is increasingly threatened by climate change, habitat fragmentation and destruction, and invasive species (Pascal, Deforges, Leguyader, & Simberloff, 2008). One invasive species of particular concern is the red‐vented bulbul, which is currently expanding its range out of the urbanized areas around Nouméa (the capital) where it was first introduced and where, until recently, it was restricted (Thibault, Vidal, Potter, Sanchez, & Brescia, 2018). Concerns about the range expansion of this species derive from its ability to disperse non‐native plant seeds more than native ones. It feeds predominantly on fruits (Brooks, 2013; Islam & Williams, 2000) and can consume leaves, flowers, and fruits of a large variety of species (Thibault, Vidal, Potter, Dyer, & Brescia, 2018), leading to significant impacts on agriculture and horticulture (Cummings, Mason, Otis, Davis, & Ohashi, 1994; Vander Velde, 2002; Walker, 2008). In its alien range, it has displayed a preference for numerous non‐native plant species (Sherman & Fall, 2010; Spotswood et al., 2013), and it is able to defend preferred food resources from other frugivorous avifauna (Thibault, Martin, Penloup, & Meyer, 2002). Its recent dispersal out of urbanized habitats raises questions about its potential to disperse seeds of noxious plant species along urban corridors and beyond.

Here, we combined a suite of methods to characterize the association between the red‐vented bulbul and non‐native plant species of New Caledonia and to assess the capacity of the red‐vented bulbul to disperse viable seeds from periurban habitats. We conducted gut content analysis of shot and trapped birds to quantitatively assess the varieties of fruit consumed by the bulbuls. We then determined gut passage times for favored fruits and tested, for two species, the effects of ingestion on seed germination rates. Finally, we radio‐tracked red‐vented bulbuls and used those data to predict median and maximum dispersal distances based on how far the birds flew during periods equivalent to gut transit times. Results are discussed with regard to the current range expansion of the red‐vented bulbul, and their relevance to a broader understanding of the mechanisms and impacts of seed dispersal by non‐native avian frugivores.

## 2 METHODS

### 2.1 Gut content analysis

Due to their pest status, both shooting and trapping of red‐vented bulbuls are authorized under New Caledonian Southern Province law (DEPS, 2016). In June 2016, we distributed a “call for participation” to the local hunting federation to collect bulbul cadavers from different locations within its local range. Each cadaver was frozen and labeled with the date it was shot and location details.

Gut content analysis was conducted on 139 dead bulbuls from 14 periurban habitats over 10 months around Nouméa to check for fruit and seed remains. Gastrointestinal tracts were excised, and the contents removed and washed with tap water through a 0.2‐mm sieve. The retained contents were placed in a petri dish filled with 70% alcohol and examined under a dissecting microscope at 10× magnification (Olympus SZ61). Each new item was photographed (ToupCam UCMOS camera and ToupView software) for subsequent identification and preserved in a reference collection (Lopes, Fernandes, & Marini, 2013).

Fruit and seed species identifications were made by reference to specimens in the New Caledonian Agronomic Institute's (I.A.C) seed bank and by expert botanists when matching samples were not available. Numbers of occurrences were counted, and frequency of occurrence was calculated for each different item. The frequency of occurrence corresponded to the number of samples that contained the item divided by the total number of samples. We then related these frequencies to the plant distribution status and use, to determine potential impacts of their consumption by the red‐vented bulbul.

### 2.2 Gut transit time experiment

We used bulbul individuals that were trapped between January and May 2016 and kept in an aviary. For our experiment, we randomly selected 16 individuals that were placed in numbered individual bird cages. Each cage had the same volume of approximately 0.25 m^3^ and was equipped with a perch and water dispenser (Linnebjerg, Hansen, & Olesen, 2009). Bulbuls were fed ad libitum with a mix of chicken grain, nectar powder, and water. To avoid any bias in the measurement of retention times due to birds’ stress in confinement conditions (Afik & Karasov, 1995), birds were maintained in individual cages for 2 weeks before initiating the experiment.

The usual supply of food was removed from each cage at least 3 hr before each experimental session. At the beginning of each test, a variety of fruit (see below) was placed in a Petri dish inside four bulbul cages. We conducted our seed retention experiments with four types of fruit from four different plant species with different distributions. Plant species were selected based on (1) direct observations of consumption, data from the literature, and results of the diet study; (2) their conservation value; and (3) their seasonal availability and morphological characteristics (small red fruit being preferred). We used berries of the endemic shrub *Myrtastrum rufopunctatum* (Pancher ex Brongniart et Gris), fruit of the native tree *Ficus prolixa* G. Forst., berries of the introduced vine *Passiflora suberosa* L., and berries of the invasive shrub *Schinus terebinthifolius* Raddi (Table S1). We did not select *Solanum torvum* and *Syzygium cuminin,* two of the most consumed species*,* because they were not available in sufficient numbers in the field at the time of the experiment. Each bulbul was tested with a different fruit species and four bulbuls were tested simultaneously, so that four fruit species were tested simultaneously. Two observers, hidden behind a bulkhead, noted the time and number of items consumed and defecated for 60 min (Schabacker & Curio, 2000). Defecated seeds were then collected and stored for a maximum of 24 hr in empty Eppendorf tubes until planting, to avoid any modification of the germination capacity.

We first controlled for the equivalent palatability of the four fruit species for the bulbuls. To do so, we calculated the mean reaction time for each fruit species. This is the time between introduction of the fruit and first fruit consumption by each individual bulbul. Reaction times are presented as mean time (s) ± standard error. We then calculated the mean gut passage time for each of the fruit species, to explore potential variations in the retention time due to specific fruit properties. In order to evaluate the dispersal capacity of the red‐vented bulbul, we also calculated the gut passage time depending on whether a fruit contained one or several seeds, following the method presented in Weir and Corlett (2007). This method suits the estimation of dispersal capacity, as it allows the estimation of three thresholds in the passage time of seeds through the gut. We calculated the median time for (1) the first defecation of multiseeded fruits, (2) the defecation of one‐seeded fruits, and (3) the last defecation of multiseeded fruits.

### 2.3 Germination test

We explored potential effects of passage through a bulbul's gut on the seed coat or endocarp of two fruit species following the approach of Samuels and Levey (2005). We compared the germination speed (time of each germination) and rate (percentage of seeds that germinated) of control‐extracted plant seeds versus defecated seeds of the two plant species that had the longest gut passage time, *M. rufopunctatum* and *S. terebinthifolius*. In a context of resource constraints, we chose these species to avoid a potential underestimation of the dispersal distance by the red‐vented bulbul. Both the control and treatment samples comprised 160 seeds extracted from fruit from an individual plant. The germination substrate contained 60% planting mold (Dalton's premium seed mix) and 40% vermiculite (Ausperl grade 2, 2–4 mm). We placed 35 planting cells, each one sowed with two seeds, on trays that were placed on warming tables (24°C) inside a glasshouse, with normal daylight conditions (approx. 11 hr of sunlight per day) and regular water supply. Cells were checked every day, and seedlings were counted and removed as soon as the hypocotyl was more than 1 mm in length. We stopped the monitoring 50 days after the last germination was recorded. Differences in germination rates between the two treatments were explored through chi‐square test of independence in R version 3.4.0 (R Development Core Team, 2017).

### 2.4 Spatial activity of bulbuls

We estimated the spatial activity of bulbuls according to the method described in Weir and Corlett (2007). This method uses three periods for activity monitoring: (1) the minimum retention time of multiseeded fruits; (2) the median retention time of one‐seeded fruits; and (3) the maximum retention time for multiseeded fruits. We calculated these three periods from the seed retention experiment and estimated the movements of bulbuls for each period. Monitoring was carried out between July 2016 and September 2016, corresponding to the cool, dry, and nonbreeding season for this species.

Adult red‐vented bulbuls were trapped using a decoy bird in an aviary trap, fitted with VHF transmitters (Titley Scientific, LT6‐337), and then released. Bird position and movements were monitored following the method described in Raim (1978). The transmitters weighted 470 mg and transmitted a pulsed signal at 150 Mhz. We tracked the VHF signal with a numeric receiver (Titley Scientific, Australis 26k), equipped with a flexible 3‐element Yagi antenna from Titley Scientific. Observations started 24 hr after release, allowing each bird time to acclimate to the tag. When a tagged bird was located, the observer followed the individual at a 20 m distance and monitored its activity. Birds were observed with binoculars, and their positions recorded using a GPS unit, at the start of each monitoring period and at each new location visited by the tagged bird during the monitoring session. The duration of a session varied from a few minutes to an hour, depending on the topography and bird activity.

Data were compiled in QGIS software (QGIS Desktop v.2.18.1; QGIS Development Team, 2017). Each displacement was calculated from a *T*_0_ location, as (1) the median of distances from the T_0_ location to all the locations visited by the bird and (2) as the largest of these distances. We did this calculation for the three “retention” periods and at every 10‐min interval. When the monitoring session was long enough, we considered the locations occupied after the studied time periods as independent *T*_0_. Constraints that justify this approximation are presented in Weir and Corlett (2007). These authors also discussed potential biases and why they are unlikely to have much impact on the estimates of median movements.

## 3 RESULTS

### 3.1 Plant consumption

We found food remains in 115 of 139 gut contents examined, and plant items were found in 93% (*n* = 107) of samples (Table 1). Seeds and fruits represented about 80% of the plant remains we found in the bulbul guts: Seeds represented nearly 37% of plant items, whereas entire fruits and fruit flesh accounted for 25% and 20% of plant items, respectively (Figure S1). The remaining 20% consisted of fruit skins (13%), leaf parts (1.8%), and flowers (0.5%). We were able to identify a minimum of 14 plant families that were eaten by the red‐vented bulbul in the Southern Province of New Caledonia (Table 1). Among these plant families, *Myrtaceae* and *Solanaceae* were the most frequent in bulbuls’ guts, corresponding to 25.2% (*n* = 29) and 12.2% (*n* = 14) of occurrence, respectively. Some remains were more intact than others, allowing the identification of 16 different items at a species level, and one to genus. Most identified taxa were non‐native (14 species); five of these are considered invasive in New Caledonia. Among these invasive species, the most important were the Turkey berry (*Solanum torvum*; 9.5%), the Persian lilac (*Melia azedarach*; 8.6%), the Guava (*Psidium guajava*; 5.2%), and the Corkystem Passionflower (*Passiflora suberosa*; 5.2%). The Brazilian peppertree (*S. terebinthifolius*) was also consumed. This pioneer evergreen shrub is listed as one of the 100 world's worst invasive species by the IUCN (Lowe, Browne, Boudjelas, & De Poorter, 2000). The most frequently consumed plant species were the non‐native Java Plum (*Syzygium cumini*; 10.3%) and *Myrtastrum rufopunctatum* (9.5%), the only endemic species identified in the diet of New Caledonian bulbuls. Of the 16 plant species identified in the bulbuls’ diet, eight species are cultivated as food plants and six as ornamentals. All of these species have red, orange, or dark purple fleshy diaspora; the berries of the Brazilian peppertree being the less fleshy. The largest seed (8 mm long) that we found intact in a bulbul stomach was from *Litchi chinensis*.

**Table 1:** Percent frequency of occurrence of plant remains present in 107 digestive tracts of red‐vented bulbuls (*Pycnonotus cafer*)

Family	Species	Frequency of occurrence (%)	*n*	Distribution status^a^
Plantae		93.0	107	
Anacardiaceae	*Schinus terebinthifolius*	0.9	1	Non‐native
Annonaceae		1.7	2	
	*Annona muricata*	0.9	1	Non‐native
	*Annona squamosa*	0.9	1	Non‐native
Araliaceae	*Schefflera actinophylla*	2.6	3	Non‐native
Arecaceae	spp.	2.6	3	
Asparagaceae	*Cordyline fruticosa*	0.9	1	Native
Meliaceae	*Melia azedarach*	**8.7**	**10**	Non‐native
Moraceae	*Ficus* sp.	5.2	6	Native
Myrtaceae		25.2	29	
	*Myrtastrum rufopunctatum*	**9.6**	**11**	Native
	*Psidium guajava*	5.2	6	Non‐native
	*Syzygium cumini*	**10.4**	**12**	Non‐native
	spp.	0.9	1	
Passifloraceae		6.1	7	
	*Passiflora foetida*	0.9	1	Non‐native
	*Passiflora suberosa*	5.2	6	Non‐native
Petiveriaceae	*Rivina humilis*	1.7	2	Non‐native
Rubiaceae	spp.	1.7	2	
Rutaceae	*Murraya paniculata*	4.3	5	Non‐native
	spp	0.9	1	
Sapindaceae	*Litchi chinensis*	2.6	3	Non‐native
Solanaceae		12.2	14	
	*Solanum torvum*	**9.6**	**11**	Non‐native
	*Solanum lycopersicum*	0.9	1	Non‐native
	spp.	1.7	2	

a Distribution status according to the Department of Environment of the Southern Province (2016).

Most frequent plant species (n>10) are bolded.

### 3.2 Seed retention times

**Figure 1:**
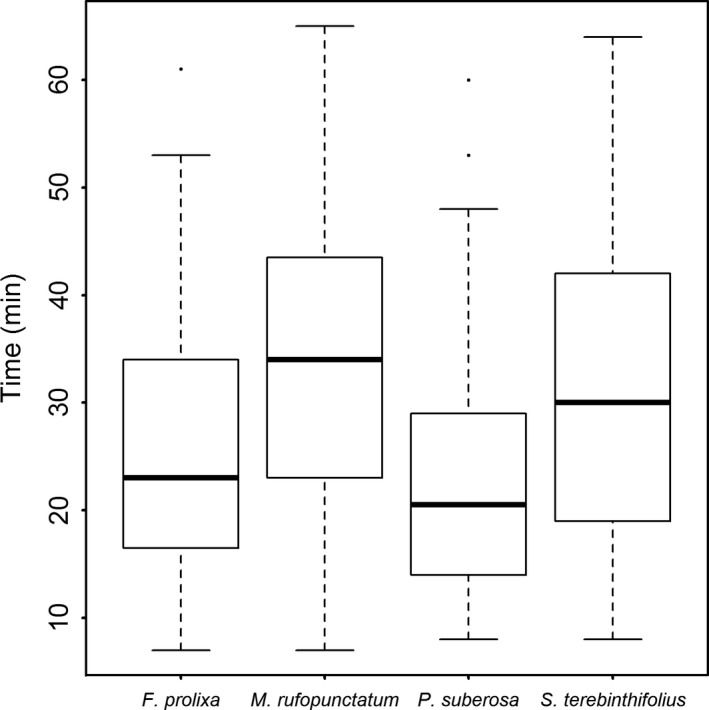
Mean digestive retention times by the red‐vented bulbul of *Ficus prolixa*,* Myrtastrum rufopunctatum*,* Passiflora prolixa*, and *Schinus terebinthifolius* seeds

There were no significant differences in the palatability of the four fruit species. Mean reaction time varied from 56 ± 34 min for *P. suberosa* to 155 ± 66 min for *S. terebinthifolius*. On average, individual bulbuls started feeding on *M. rufopunctatum* and *F. prolixa* after 123 ± 47 min and 77 ± 25 min, respectively. Mean gut passage times of the four plant species are presented in Figure 1. During our experiment, minimum and maximum retention times were of 7 and 65 min, respectively. Fruits of *P. suberosa* and *F. prolixa* were digested in 23 ± 1.13 min and 26 ± 1.35 min on average, a little faster than those of *S. terebinthifolius* (31 ± 1.45 min) and *M. rufopunctatum* (33 ± 1.83 min). Gut passage time was significantly different between the four species (ANOVA *F *=* *11.1; *df* = 3; *p *=* *6.185e^−07^). In our experiment, *M. rufopunctatum* and *S. terebinthifolius* had longer gut passage times that *F. prolixa* and *P. suberosa* (see pairwise *t* tests in Table S2).

The three gut passage thresholds estimated for the red‐vented bulbul in New Caledonia are summarized in Table 2. We conducted 49 measures of gut passage time for multiseed fruits. The first seeds took between 7 and 41 min to be dropped, with a median of 14 min. The last seeds were dropped after 13 to 65 min with a median of 41 min. We replicated the test 93 times using berries of *S. terebinthifolius* and recorded median gut passage time of 30 min, with minimum and maximum times corresponding to 8 and 64 min, respectively.

**Table 2:** Gut passage times in minutes for seeds in single‐ and many‐seeded fruits consumed by the red‐vented bulbul in New Caledonia. Many‐seeded fruits: *Myrtastrum rufopunctatum*,* Passiflora suberosa*, and *Ficus prolixa* One‐seed fruit: *Schinus terebinthifolius*

First seed, many‐seeded fruits			Single‐seeded fruits			Last seed, many‐seeded fruits		
Median	Range	*n*	Median	Range	*n*	Median	Range	*n*
14	7–41	49	30	8–64	93	41	13–65	49

### 3.3 Effect of passage through the gut on germination

**Figure 2:**
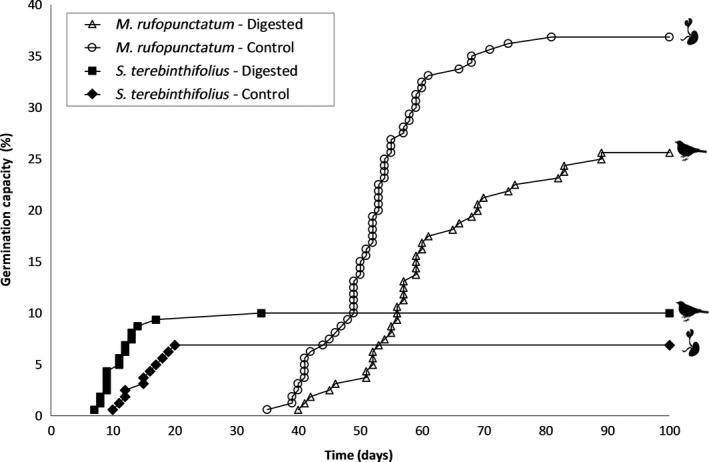
The influence of passage through the gut of a red‐vented bulbul on germination rates of *Myrtastrum rufopunctatum* and *Schinus terebinthifolius* seeds

Results of the germination tests are presented in Figure 2. Control seeds of the endemic shrub *M. rufopunctatum* reached the maximum germination rate (37% in 80 days). In comparison, only 25% of *M. rufopunctatum* seeds that passed through the gut of red‐vented bulbuls germinated. Thus, consumption by the red‐vented bulbul significantly reduced the germination rate of seeds of *M. rufopunctatum* by a factor 1.5 (χ^2^ = 4.71, *df* = 1, *p *=* *.03). Furthermore, germination of the digested seeds of this species was slightly delayed (40–89 days) compared with control seeds (35–81 days; *t *=* *−3.59, *p *=* *.0006). The germination success of the seeds of the invasive shrub *S. terebinthifolius* was very low in our experiment. Digested seeds germinated between days 7 and 17, reaching a success rate of 10% only. Germination of control seeds started a few days later (10–20 days) and only 7% of planted seeds germinated successfully, although the difference between the two treatments was not significant (χ^2^ = 1.01, *df* = 1, *p *=* *.31).

### 3.4 Dispersal capacity

**Figure 3:**
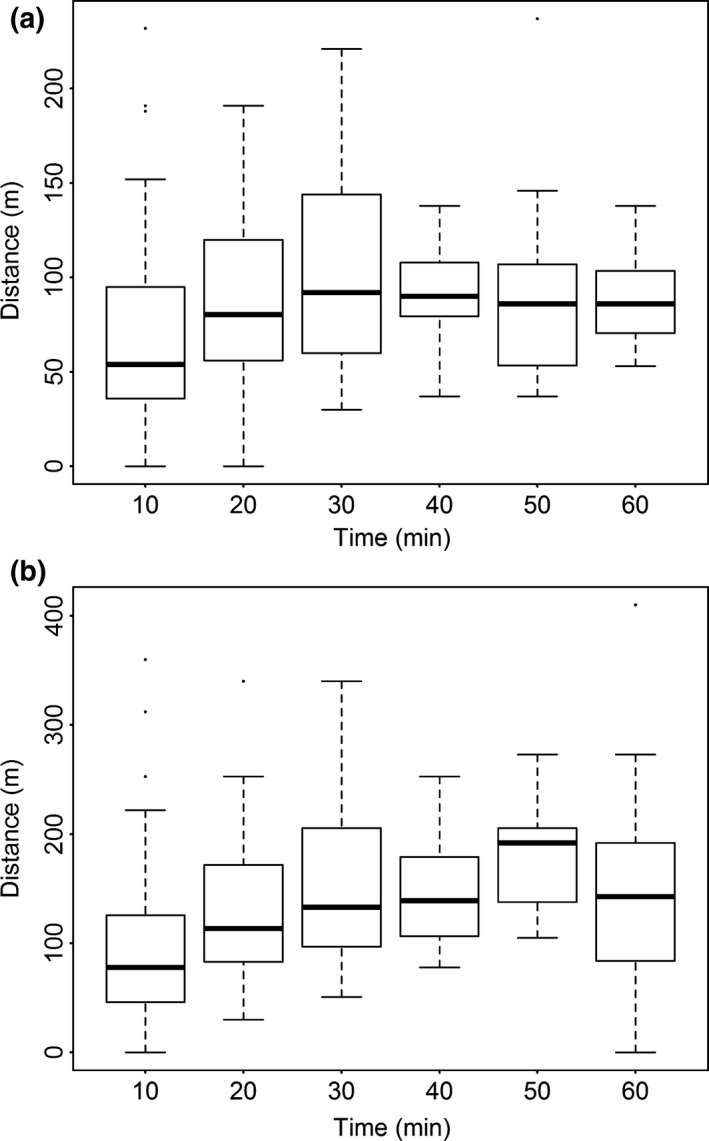
Graph of the distance travelled as a function of time for the red‐vented bulbul. Distances travelled from arbitrary starting points were determined at 10 intervals. (a) Median distance covered as a function of time. (b) Maximum distance covered as a function of time

We conducted 11 monitoring sessions of three bulbul individuals’ movements. Median distance and maximum distance travelled are presented as a function of consecutive 10‐min periods, giving an overview of dispersal capacity of the red‐vented bulbul from the feeding time to the last dropping (Figure 3). This figure suggests that, when foraging, movements of the red‐vented bulbul are restricted to a radius of 100 m around a resource tree. On average, the birds covered this distance within 30 min after feeding on a specific tree. Maximum movements recorded suggest that red‐vented bulbuls can cover up to 100 m in 20 min and up to 200 m in the 50 min following a food intake.

The median distance travelled by individual bulbuls during the three periods corresponding to our three defined gut passage times are shown in Table 3. Our results suggest that the median distance covered by red‐vented bulbuls in 14 min was approx. 70 m (*n* = 44), and it was 92 m after 41 min (*n* = 11). The median distance covered within 30 min was of 92 m (*n* = 23). According to the minimum and maximum distance we observed, we estimated that a bulbul is able to spread the seeds of multiseeded fruit over distances of up to 273 m from the source tree. However, for these fruits, the estimated median dispersal distances were 70–92 m. For single‐seeded fruit, we estimated the median dispersal distance by the red‐vented bulbul to be 92 m from the source tree (range 30 to 221 m).

**Table 3:** Displacements (m) of bulbul individuals from arbitrary starting points during the gut passage times (GPT) for seeds (i.e., 14 min; 30 min; and 41 min; see Table 2)

Displacement during the minimum GPT (m)			Displacement during the median GPT (m)		Displacement during the maximum GPTa (m)		
Median	*n*	Lowest	Median	*n*	Median	*n*	Highest
77	44	0	92	23	92	11	273

## 4 DISCUSSION

### 4.1 Preference for non‐native fruits

For now, the red‐vented bulbul remains restricted to man‐modified habitats in New Caledonia (Thibault, Vidal, Potter, Sanchez, et al., 2018) where ornamental plant species are often disproportionally composed of non‐native plant species (Smith, Thompson, Hodgson, Warren, & Gaston, 2006). The diversity of plant material found in the intestines we analyzed confirmed that the red‐vented bulbul feeds on a large variety of plant structures and species (Walker, 2008). We also confirmed an expected association between the bulbul and a community of non‐native plant species, with more than 80% of the consumed plant species identified being exotic to New Caledonia. This suggests that consumption of introduced plant species fairly meets the daily energetic requirement of the red‐vented bulbuls, as demonstrated in a South African bulbul species (Jordaan, Johnson, & Downs, 2011). We confirmed that red‐vented bulbuls consume the fruit of the invasive *S. terebinthifolius*, although the frequency of occurrence in the gut samples was low. The bulbuls’ contribution to the dispersal of *S. terebinthifolius* along roads and urban corridors might thus rely on other factors such as fruit phenology and availability (Leck, 1972). Their diet within their natural range suggests that the bulbuls may prefer fleshier fruits (Patyal & Rana, 2003; Rana, Narang, & Patyal, 2005). Our data suggest that the bulbuls contribute to the dispersal of two other exotic species, *R. humilis* and *P. suberosa*. Fruits of these species are small, round, and fleshy and are red/dark purple in color. These appear to be characteristics of preferred fruit for the red‐vented bulbul (Spotswood et al., 2013). All of the consumed fruit species we identified here have red fleshy diaspore, including fruit of the shrub *M. rufopunctatum* that occurred frequently in bulbul gut samples. *M. rufopunctatum* is endemic to New Caledonia and promoted as an ornamental and revegetation plant (Gâteblé, 2016), so this may be one native species that might benefit from consumption by the red‐vented bulbul if it remains viable following passage through the gut. Our germination data (discussed below) indicated that this was not the case, with passage through the gut of the red‐vented bulbul significantly reducing germination rates of *M. rufopunctatum* seeds. We included *M. rufopunctatum*, along with the invasive *S. terebinthifolius*, the introduced *P. suberosa* and the native *F. prolixa, in* the gut passage time analysis.

### 4.2 Rapid gut passage times

Passage through the gut of an animal plays a crucial role in seed dispersal and potential dispersal distance (Fukui, 1996; Proctor, 1968), but seeds can be affected differently depending on which species consume and digest it (Nogales, Nieves, Illera, Padilla, & Traveset, 2005). For example, in New Caledonia, native flying foxes and pigeons are far better small‐ and medium‐sized seed dispersal agents than introduced rodents, with rodents nibbled the seeds whereas pigeons and flying foxes swallowed them whole, resulting in significantly higher germination success (Duron, Garcia‐Iriarte, Brescia, & Vidal, 2017). However, the chemical or mechanical impacts of digestion by two different bird species, although taxonomically close, can produce opposite effects on seeds germination success (Bartuszevige & Gorchov, 2006). According to the gut retention time hypothesis, secondary metabolites of passerine‐mediated fruits species could act as laxatives, leading to rapid passage times (Cipollini, 2000). This phenomenon is expected to increase the rate of food intake by the bird.

In designing our experimental conditions for determination of gut passage times, we avoided potential complications with fruit palatability, bird stress, and degree of hunger by supplying the birds with fruits they are known to eat, providing an acclimation period, and removing their normal food for a set period before each trial (Levey & Karasov, 1992). All individual bulbuls that ate during the experiment started pecking fruits within the first minute of the experiment, allowing direct comparisons of the results obtained with different fruits and across individuals. The gut passage times we measured for the red‐vented bulbuls were consistent with results of a previous study conducted with 11 fruit species digested by the white‐spectacled bulbul, *P. xanthopygos,* in Israel (Barnea, Yom‐Tov, & Friedman, 1991). Gut passage times ranged from 7 to 65 min, with averaged values of 23 to 32 min depending on the fruit species. Barnea et al. (1991) reported gut passage times of 9 to 33 min depending on the fruit species. Linnebjerg et al. (2009) reported slightly shorter values in red‐whiskered bulbuls, *P. jocosus*, of Mauritius with gut passage times around 15 min. Such differences in gut passage times are related in part to the specific characteristics of each fruit (Traveset, 1998). This held true for our experiment, as we selected fruit from different species but with very similar characteristics and found small but significant differences in their mean retention times. These differences can be partly explained by the digestion physiology of the bird (Afik & Karasov, 1995) and the flesh structure (Levey, 1986), with juicy fruits (*P. suberosa*,* F. prolixa*) being digested more rapidly than firm fruits (*S. terebinthifolius*). Our measurements of median retention times for single versus multiseeded fruits were comparable to the results Weir and Corlett (2007) obtained for the light vented bulbul, *P. sinensis*, and for the red‐whiskered bulbul, in tropical landscapes of China.

### 4.3 Nonhomogeneous impacts on germination

The impact of passage through the gut of a red‐vented bulbul on germination rates differed between the two fruit species we tested, with germination success of *M. rufopunctatum* seeds being significantly lower when they were collected from bulbul droppings compared with control seeds that had been extracted from their fruits, but the reverse was true for *S. terebinthifolius*. Negative effects of gut passage are typically due to damage caused to the seed coat or exocarp (Samuels & Levey, 2005), and our data imply that the red‐vented bulbul is not an effective disperser of *M. rufopunctatum* seeds. Whether the negative effect observed here for *M. rufopunctatum* seeds is indicative of the effects of passage through the gut on germination rates of other native species should be investigated, ideally in a comparative study that also assesses the effects of passage through the intestinal tract of native bird species.

In contrast to the negative effects of passage through the gut of a red‐vented bulbul on germination rates of *M. rufopunctatum* seeds, germination success of *S. terebinthifolius* seeds did not differ significantly between treatment and control seeds for which exocarp had been removed. Panetta and McKee (1997) obtained similar results with 22 other bird species. Seeds digested by red‐vented bulbuls had a slightly higher germination rate and speed compared with control seeds. However, the success of germination for this species under glasshouse conditions (approx. 10%), even if consistent with results of Nilsen and Muller (1980), did not allow a statistical test of these differences. Dormancy lifting mechanisms such as chemical scarification are known to enhance the germination of *S. terebinthifolius* (Ewel, Ojima, Karl, & DeBusk, 1982). Here, we showed that the digestion of *S. terebinthifolius* by the red‐vented bulbul had a similar effect on seeds germination as removal of the exocarp. From previous studies, we know that removal of the exocarp of *S. terebinthifolius* by frugivores promotes germination (*Zosterops lateralis*; Panetta & McKee, 1997). Therefore, we suggest that consumption by the red‐vented bulbul also promote the germination success of *S. terebinthifolius*. Once again, this observation is consistent with the hypothesis of a strong mutualistic relationship between introduced red‐vented bulbuls and non‐native plant communities. Benefits from the fruit consumption by the bird, and from the dispersal by the fruit, could favor the expansion of both non‐native species.

### 4.4 Short‐distance dispersal

By combining gut transit time data with bird movement data, we predict that the maximum distances that red‐vented bulbuls would likely distribute these fruit from source trees are 150 m. Similar seed dispersal distances have been reported for adults red‐whiskered bulbuls (Weir & Corlett, 2007) and white‐spectacled bulbuls (*P. xanthopygos*; Spiegel & Nathan, 2007). Our results were also consistent with Jordano, Garcia, Godoy, and García‐Castaño's (2007) that small passerine species are predominant short‐distance (<250 m) seed dispersers, although differences in species, habitat, and season‐dependent resource availability prevent direct comparisons across studies. Laver and Kelly (2008) rightly warned about the risks associated with such comparison, using the case of estimations of home range size in different studies. Nevertheless, we believe that the similarities between the ranges of our results and those of previous studies on pycnonotid species add confidence to our assessment of the small‐distance dispersal capacity of the red‐vented bulbul in periurban habitats. Exploring the contribution of biological factors such as bird maturity, sex, reproductive status, habitat, resources availability, and distance from the invasion front could help improve estimation of the seed dispersal capabilities of the red‐vented bulbul.

### 4.5 Seed dispersal effectiveness of the red‐vented bulbul

Even given the red‐vented bulbul's apparently short‐distance seed dispersal capabilities, the widespread planting of some of its preferred non‐native fruit species in periurban environments may aid its range expansion north and south of Nouméa and further disperse the seeds of these exotic plants, potentially driving an “invasional meltdown”. The study highlights the need for further research on the effects that consumption by red‐vented bulbuls has on germination rates of other fruit‐bearing plants species, both native and invasive, and how its seed dispersal capabilities compare with those of native frugivorous birds. Particular attention should be paid to the bulbul's feeding seasonality and intensity on native and non‐native plants species, including *Miconia calvescens* (Meyer, 1996). This will aid the design appropriate and effective conservation actions throughout the red‐vented bulbul's alien distribution.

## 5 CONCLUSION

We present an overview of various context‐dependent and distance‐dependent mechanisms that contribute to the seed dispersal capabilities of the invasive red‐vented bulbul. These bulbuls showed preference for non‐native fruits species in the study areas. This, combined with enhanced germination rates following gut transit of seeds of the highly invasive *S. terebinthifolius,* could represent an example of “invasional meltdown”. Such a mutualistic relationship could lead to major conservation issues, particularly in ecosystems that host a large number of endemic plant species such as the ultramafic maquis of New Caledonia. Therefore, we suggest that the population of red‐vented bulbuls should be confined to its current distribution range in man‐modified habitats of the Southern Province until a dedicated country‐scale management strategy is designed. Similarly, the small population of *M. calvescens* should be confined and/or eradicated, and substantial survey effort should be dedicated to prevent any overlap between this invasive plant and red‐vented bulbuls. Finally, there is an urgent need for research programs dedicated to describe dispersal of seeds by native frugivores in New Caledonia to prevent both future threats on endemic species by introduced frugivores and prevent the dispersal of non‐native plants out of inhabited areas.

## CONFLICT OF INTEREST

None declared.

## AUTHORS’ CONTRIBUTION

Martin Thibault, Felix Massé, Fabrice Brescia, and Bruno Fogliani conceived the study. Martin Thibault, Felix Massé, and Aurore Pujapujane contributed to data curation. Martin Thibault performed formal analysis. Fabrice Brescia and Bruno Fogliani acquired funding. Martin Thibault, Felix Massé, Aurore Pujapujane, Laurent Bordez, and Guillaume Lannuzel carried out investigation. Martin Thibault, Félix Massé, Laurent Bordez, Bruno Fogliani, and Guillaume Lannuzel contributed to methodology. Martin Thibault, Felix Massé, and Fabrice Brescia administered the project. Fabrice Brescia and Bruno Fogliani collected the resources. Fabrice Brescia, Murray Alan Potter, Eric Vidal, and Bruno Fogliani supervised the study and contributed to validation and visualization. Martin Thibault wrote the original draft of the manuscript. Martin Thibault, Laurent Bordez, Felix Massé, Aurore Pujapujane, Guillaume Lannuzel, Bruno Fogliani, Eric Vidal, Murray Alan Potter, and Fabrice Brescia wrote, edited, and reviewed the manuscript.
