# Supplementary material for: “Liaisons dangereuses”: The invasive red‐vented bulbul (Pycnonotus cafer), a disperser of exotic plant species in New Caledonia
Source: Ecol Evol. 2018 Aug 24;8(18):9259–69. doi: 10.1002/ece3.4140 (PMC6194277; doi:10.1002/ece3.4140)

**Figure S1**. Number and frequency of the different plant parts found in the digestive tracts of 115 red-vented bulbuls.


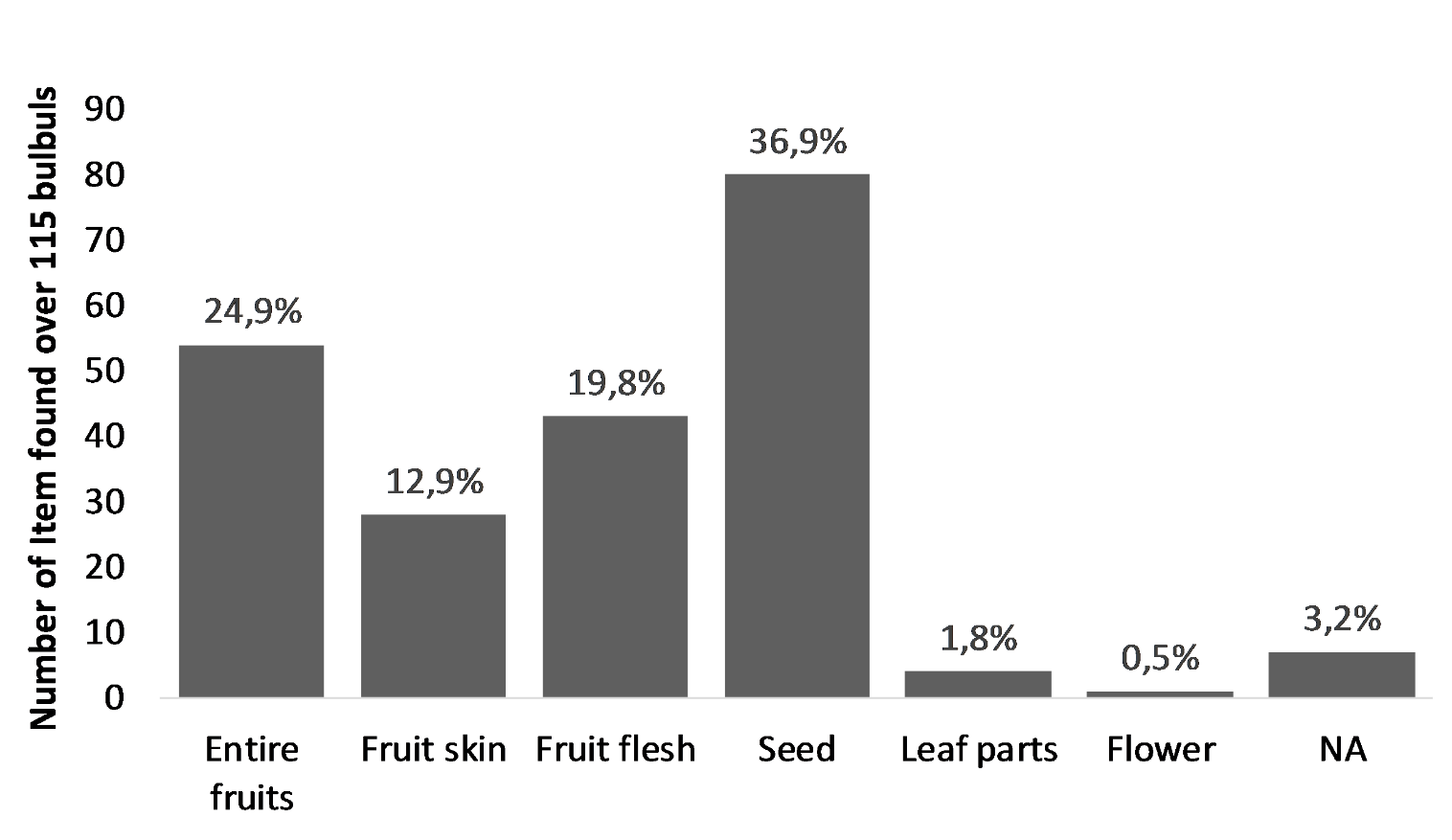

Supplement: Supplementary file 1 [file ECE3-8-9259-s001.docx]
