# Supplementary material for: “Liaisons dangereuses”: The invasive red‐vented bulbul (Pycnonotus cafer), a disperser of exotic plant species in New Caledonia
Source: Ecol Evol. 2018 Aug 24;8(18):9259–69. doi: 10.1002/ece3.4140 (PMC6194277; doi:10.1002/ece3.4140)
Supplement: Supplementary file 2 [file ECE3-8-9259-s002.docx]

**Table S1.** Overview of the main characteristics of the four fruit species used in the study.

|  |  |  |  |  |
| --- | --- | --- | --- | --- |
|  | ***S. terebintifolius*** | ***M. rufopunctatum*** | ***P. suberosa*** | ***F. prolixa*** |
| Type | berry | berry | berry | figs |
| Color | red | purple | purple | red-orange |
| Shape | spherical | egg-shaped | spherical | spherical |
| Diameter (cm) | 0.4-0.5 | 1 | 0.6-1.9 | 1 |
| Seeds (n) | 1 | > 30 | >20 | >100 |
| Plant type | shrub | shrub | liana | tree |
| Habitat | generalist | maquis* | drained | coasts. forests |
| Conservation | invasive | endemic | introduced | native |
| Fructification | Apr-Jun | Aug-Dec | May-Dec | May-Jun |
| Uses | ornamental food. medicinal. | ornamental. revegetation | medicinal | wood. latex |
| Reference | *GEE. 2012* | *Gâteblé. 2006* | *GEE. 2012* | *Suprin. 2011* |
|  |  |  |  |  |
| **maquis: shrubby vegetation growing on ultramafic outcrops* | | | |  |
