# Supplementary material for: “Liaisons dangereuses”: The invasive red‐vented bulbul (Pycnonotus cafer), a disperser of exotic plant species in New Caledonia
Source: Ecol Evol. 2018 Aug 24;8(18):9259–69. doi: 10.1002/ece3.4140 (PMC6194277; doi:10.1002/ece3.4140)
Supplement: Supplementary file 3 [file ECE3-8-9259-s003.docx]

**Table S2**. P values of pairwise Student’s t-tests exploring the differences in gut passage times of *Ficus prolixa*. *Myrtastrum rufopunctatum*. *Passiflora prolixa* and *Schinus terebinthifolius*.

|  | *F. prolixa* | *M. rufopunctatum* | *P. suberosa* |
| --- | --- | --- | --- |
| *M. rufopunctatum* | 0.0043 | - | - |
| *P. suberosa* | 0.1774 | 7.30 E-06 | - |
| *S. terebinthifolius* | 0.0238 | 0.3728 | 5.00 E-05 |
